# Comparison of Multiple Radiomics Models for Identifying Histological Grade of Pancreatic Ductal Adenocarcinoma Preoperatively Based on Multiphasic Contrast-Enhanced Computed Tomography: A Two-Center Study in Southwest China

**DOI:** 10.3390/diagnostics12081915

**Published:** 2022-08-08

**Authors:** Hongfan Liao, Yongmei Li, Yaying Yang, Huan Liu, Jiao Zhang, Hongwei Liang, Gaowu Yan, Yanbing Liu

**Affiliations:** 1 College of Medical Informatics of Chongqing Medical University, No. 1 Yixueyuan Road, Yuzhong District, Chongqing 400016, China; 2 Department of Radiology, The First Affiliated Hospital of Chongqing Medical University, Chongqing 400016, China; 3 Department of Pathology, Molecular Medicine and Cancer Research Center, Chongqing Medical University, Chongqing 400016, China; 4 GE Healthcare, Shanghai 201203, China; 5 Department of Radiology, The Third Affiliated Hospital of Chongqing Medical University, Chongqing 401120, China; 6 Department of Radiology, Suining Central Hospital, Suining 429000, China

**Keywords:** pancreatic ductal adenocarcinoma, histological grade, radiomics, machine learning, prognosis

## Abstract

Background: We designed and validated the value of multiple radiomics models for diagnosing histological grade of pancreatic ductal adenocarcinoma (PDAC), holding a promise of assisting in precision medicine and providing clinical therapeutic strategies. Methods: 198 PDAC patients receiving surgical resection and pathological confirmation were enrolled and classified as 117 low-grade PDAC and 81 high-grade PDAC group. An external validation group was used to assess models’ performance. Available radiomics features were selected using GBDT algorithm on the basis of the arterial and venous phases, respectively. Five different machine learning models were built including k-nearest neighbour, logistic regression, naive bayes model, support vector machine, and random forest using ten times tenfold cross-validation. Multivariable logistic regression analysis was applied to establish clinical model and combined model. The models’ performance was assessed according to its predictive performance, calibration curves, and decision curves. A nomogram was established for visualization. Survival analysis was conducted for stratifying the overall survival prior to treatment. Results: In the training group, the RF model demonstrated the optimal predictive ability and robustness with an AUC of 0.943; the SVM model achieved the secondary performance, followed by Bayes model. In the external validation group, these three models (Bayes, RF, SVM) also achieved the top three predictive ability. A clinical model was built by selected clinical features with an AUC of 0.728, and combined model was established by an RF model and a clinical model with an AUC of 0.961. The log-rank test revealed that the low-grade group survived longer than the high-grade group. Conclusions: The multiphasic CECT radiomics models offered an accurate and noninvasive perspective to differentiate histological grade in PDAC and advantages of machine learning models including RF, SVM and Bayes were more remarkable.

## 1. Introduction

Pancreatic ductal adenocarcinoma (PDAC) is a notoriously digestive carcinoma due to its occult onset and invasive progression [1]. Despite advancements in medical technology, the PDAC patients remained poor prognosis with five-year overall survival rate as low as 7% [2,3,4]. Early surgical resection is the only curative treatment modality for PDAC [5]. Therefore, it is extremely crucial to make an accurate diagnosis and recognize the patients who can benefit from surgical resection, helping clinical physicians select available treatment protocols.

Histological grade is considered as a significant independent factor in PDAC, and low-grade PDAC is associated with longer postoperative survival [6,7], while high-grade PDAC is inclined to predict shorter survival and higher mortality [8]. Furthermore, high-grade PDAC do not have an obviously improved prognosis after radical surgery, and these patients could be influenced by postoperative complications such as pancreatitis and pancreatic fistula, and have to endure an awful quality of living [9,10]. Previous studies have reported [11,12,13], combining serum tumor biomarkers and invasive CT manifestation, high-grade PDAC is prone to receive chemotherapy, radiotherapy and palliative therapy, and low-grade PDAC without invasive signs was suggested to undergo operation as early as possible. Currently, the histological grade of PDAC is merely evaluated by postoperative specimen which is highly invasive and may delay the choice of available therapeutic protocol [14,15]. Moreover, the sample obtained by puncture biopsy is not accurate because it couldn’t represent the whole tumor due to heterogeneity in time and space. Thus, one precise and preoperative diagnosis tool is necessary for realizing precision medicine.

Radiomics, as an emerging discipline, can non-invasively transform medical images into multidimensional, potential information through quantizing features that are imperceptible to the naked eyes, and explore features’ associations with pathophysiological changes [16,17,18]. Recently, radiomics analysis in the brain, lung, pancreas and prostate is widely applied with the progress of state-of-the-art informatic technology, aiming to achieving computer-aided-diagnosis and providing clinical support in the decision-making process [19,20,21,22]. Past studies denoted the success of radiomics depend on interpretability, repeatability and reproductibility of constructed models. However, there existed no reports about comparison and investigation in choosing machine learning approaches about the histological grade in PDAC to our knowledge. Thus, it is essential and significative to provide evidence in selecting appropriate models for solving clinical problems. In this study, we compare five different machine learning approaches to construct and verify CECT-based radiomics model for distinguishing low-grade and high-grade PDAC.

## 2. Materials and Methods

### 2.1. Patients

**Figure 1:**
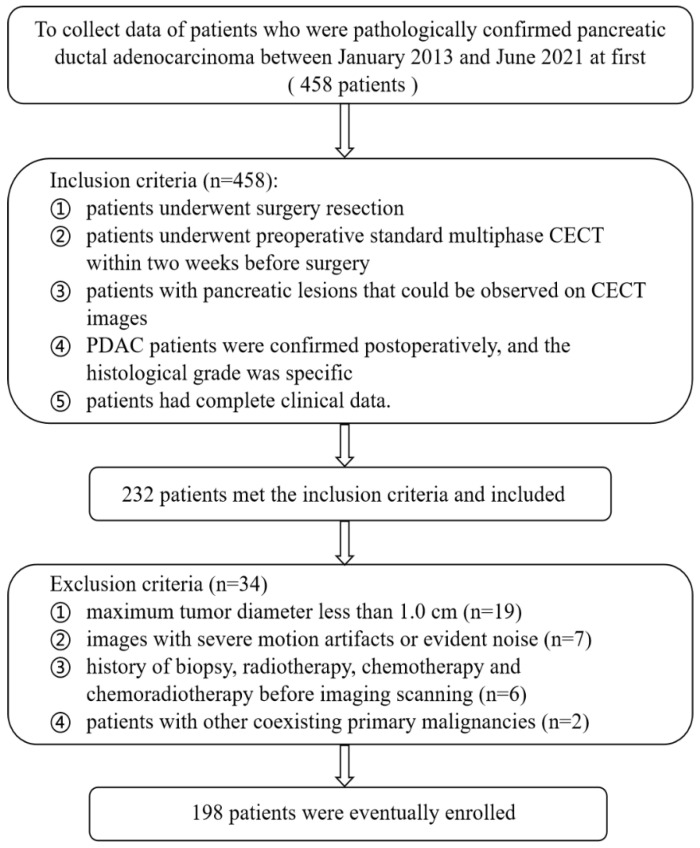
Flowchart for selecting the study population in internal group.

This research was authorized by the local ethics organization of the first affiliated hospital of Chongqing medical university (No: 2022-63), and informed consent was needless due to the retrospective nature. A total of 198 patients with primary PDAC receiving radical resection from January 2013 to June 2021 were enrolled as the internal training group. The external validation group from the third affiliated hospital of Chongqing medical university was consecutively collected from January 2022 to June 2022. The same inclusion standard between two centers was as follows: (1) patients underwent preoperative standard multiphasic CECT within two weeks before surgery; (2) pancreatic lesions should be observed visually through medical images; (3) PDAC patients were confirmed postoperatively, and the histological grade was specific; (4) patients had intact clinical data. The exclusion criteria were as follows: (1) tumor diameter is less than 10 mm; (2) serious motion artifacts or distinct noise existed; (3) treatment history of biopsy, radiotherapy, chemotherapy and chemoradiotherapy before imaging scanning; (4) other coexisting primary malignancies existed. The screened flowchart in internal center is described in Figure 1.

### 2.2. Histological Grading

All surgically removed tumors were sliced into sections, and each section was formalin-fixed and paraffin-embedded. After that, every section was cropped as slides and stained with hematoxylin-eosin. Referring the 2019 WHO classification of the digestive system tumors [23,24], the histological grade of PDAC was classified as follows: well differentiated, moderately differentiated, and poorly differentiated. Considering the imbalance of differentiated types and the similarity of biological behavior, well differentiated and moderately differentiated cases were included in the low-grade group. In contrast, poorly differentiated cases were set as the high-grade group. This dichotomous method was regarded as reflecting the pathological features and tumor invasiveness, which could better represent degree of malignancy and predict prognosis. Moreover, this classification standard was consistent with that of Chang, Qiu and Wasif et al. in dividing differentiated types [24,25,26].

### 2.3. Image Acquisition

One 128-slice multidetector-row CT (SOMATOM Definition Flash, Siemens Healthineers, Berlin, Germany) scanning was performed. The CT scanning parameters were set as follows: 120 kV; 300 mA; 0.7 pitch; collimation, 128 × 0.6 mm; beam collimation, 160 × 0.5 mm; matrix, 512 × 512; and gantry rotation time, 0.5 s. First, patients received unenhanced scanning. Second, nonionic contrast agent (Ultravist 370, Bayer Schering Pharma, Leverkusen, Germany) was injected at a dose of 1.2 mL/kg and at a flow rate of 3.5 mL/s into the antecubital vein via a pump injector (Medrad Mark V plus, Bayer, Leverkusen, Germany). The pipe was flushed by 30–40 mL normal saline. Third, 15 s after the abdominal aorta reaching 100 HU, arterial phase (AP) imaging was scanned. 30 s after the AP scanning, venous phase (VP) scanning was performed.

### 2.4. Data Collection and Follow-Up

Images data and baseline information were acquired from the institutional database. All patients were evaluated by two radiologists with 8 and 10 years of experience following double-blind principle. A consensus was reached when different ideas existed. A total of twenty-two clinical, pathological, images and laboratory characteristics were analyzed for all patients and were classified as follows: (1) clinical characteristics: gender, age, abdominal pain, backache, pancreatitis, jaundice, operation method; (2) pathological characteristics: lymph node metastasis, duodenal invasion, surgical margin status, perineural invasion; (3) images characteristics: CT-reported tumor size, tumor location, tumor density, clinical T stage, distant metastasis, parenchymal atrophy, pancreatic duct dilatation, and common bile duct dilatation; (4) laboratory characteristics: carcino-embryonic antigen (CEA) level, carbohydrate antigen 19-9 (CA19-9) level, and total bilirubin (TBIL) level. Detailed characteristics description is written in the Supplementary Material. The overall survival of PDAC patients was obtained via clinical follow-up and telephone inquiry. Overall survival was counted from the date of surgery to the time of death or the end of follow-up (1 April 2022). Deaths were set as events, whilst other situations were set as censored observations.

### 2.5. Tumor Segmentation

Manual segmentation was executed on AP and VP images, respectively. Two radiologists with 8 and 10 years of diagnostic experience, respectively, drew the outline of tumor boundary via ITK-SNAP software layer-by-layer on 5 mm CT images for every patient, excluding cystic, necrotic, blood vessels and lymph nodes. The non-enhanced phase was not selected due to tumor boundary could not visualize well between lesion and normal parenchyma. One radiologist with 10 years of experience resegmented the tumor outline for randomly selected 50 patients one month later. The delayed phase was not enrolled by reasons of the patients we selected were maximum tumor diameter at least 10 mm on CT images and lesion enhancement manifestation is similar between the venous phase and the delayed phase. In addition, some patients did not receive delayed phase scanning for reducing radiation dose if the diagnosis is fairly clear. Standard abdominal parameters were set as 250 HU of window width and 45 HU of window level. The interobserver and intraobserver reproducibility were evaluated by the intraclass correlation coefficient (ICC); Two-way random-effects model was selected for ICC analysis, and an ICC value with good consistency and reproducibility was regarded as greater than 0.75.

### 2.6. Radiomics Feature Extraction and Selection

**Figure 2:**
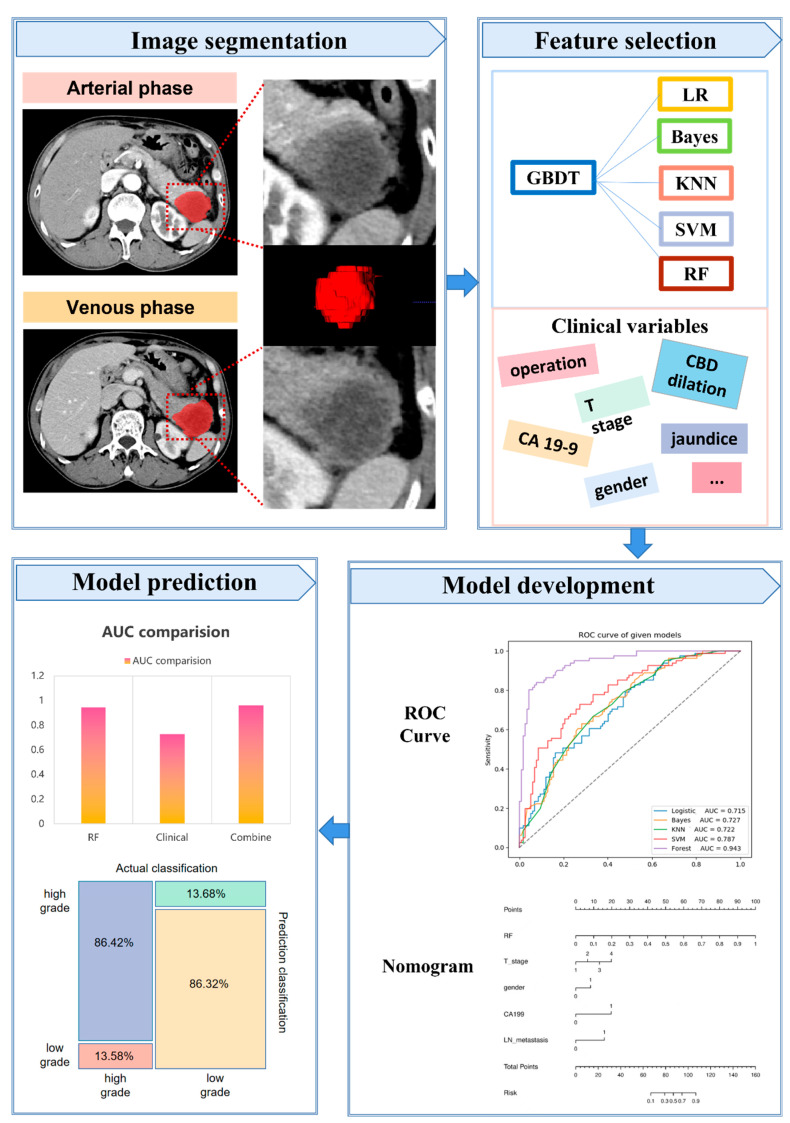
The workflow of radiomics analysis.

Images data were resampled to a uniform voxel spacing (1.0 × 1.0 × 1.0 mm^3^). The feature extraction procedure was executed on the basis of the Image Biomarker Standardization Initiative (IBSI) via the Pyradiomics package (accessed on 10 April 2022, http://www.radiomics.io/pyradiomics.html) in Python (version 3.6). Radiomics features were divided as two types including original feature and through filter transformation. The former enrolled first-order features, shape features, grey-level cooccurrence matrix features (GLCM), grey-level dependence matrix features (GLDM), grey-level run-length matrix features (GLRLM), grey-level size-zone matrix features (GLSZM), and neighbourhood grey-zone difference matrix features (NGZDM). The latter enrolled logarithm, exponential, gradient, square, square root, lbp-2D, and wavelet transformation. The workflow of radiomics analysis is showed in Figure 2.

First, feature values were cleaned and organized, including abnormal and missing feature values were replaced with median values. Data were unified by z-score standardization. After ICC analysis, the features were retained with ICC scores of more than 0.75. Second, a univariate logistic regression analysis was performed. Only the features with statistical significance (*p* value < 0.05) were selected. Then, the gradient boosting decision tree (GBDT) algorithm was chosen in order to rank the features based on the importance across all of the decision trees [27]. GBDT algorithm is derived from boosting ensemble learning, which has strong interpretability and good application effect in data mining, computational system and other fields. GBDT output the relative importance of features used by the model after model training, specifically, the global importance of features is measured by the average value of the importance of features in a single tree. The features possessing the greatest importance were ensured when the loss function-the mean square error reached minimum. The final selected features were plotted as heatmap for intuitively observing the overall expression pattern of features between different groups. The radiomics features used for building radiomics models were consistent in the internal training group and the external validation group.

### 2.7. Radiomics Model, Clinical Model and Combined Model Building and Evaluation

To construct multiple radiomics models that have the greatest ability of recognizing tumor inherent characteristics and to compare application effect of different machine learning-based models, our study chose five mainstream machine learning algorithm, including logistic regression (LR), k-nearest neighbor (KNN), naive bayes model (Bayes), support vector machine (SVM) and random forest (RF). Model building were executed using the scikit-learn (accessed on 12 April 2022, https://scikit-learn.org/stable/index.html) library. According to the Transparent Reporting of a Multivariable Prediction Model for Individual Prognosis or Diagnosis (TRIPOD) criterion [28], the models’ performance was evaluated based on ten times tenfold cross-validation. The quality of tenfold cross-validation is not lower than that of the hold-out method.

The predictive ability of the five models were assessed by the area under the curve (AUC), accuracy, sensitivity, specificity, f1 score, and recall. The delong test was implemented to assess the statistical discrepancy among AUCs. The decision curves were evaluated by measuring the net benefits with different threshold probabilities to exhibit the models’ clinical efficiency in tumor classification task. The calibration curve was used to fit the actual and predicted incidence rates. Univariate and multivariate logistic regression analysis were performed on a total of twenty-two predictor variables. Simultaneously, we performed the multicollinearity check for all variables. Ultimately, statistically significant features were selected for clinical model development. The clinical variables building clinical model is consistent in the internal training group and the external validation group. The sole best radiomics model in five radiomics models was selected and integrated with the clinical model to construct a combined model. To enhance the interpretability of the combined model, a quantitative model was built via regression coefficients and visualized as a radiomics nomogram. In general, the radiomics model, clinical model and combined model are different in the building procedure. The radiomics model adopted machine learning approaches, whereas the clinical model and the combined model used linear logistic regression.

### 2.8. Statistical Analysis

SPSS software (version 25.0), R software (version 3.6.1) and Python software (version 3.7.0) were applied for statistical analysis. Continuous variables with normal distributions are computed using independent-sample t tests; continuous variables with non normal distributions were used wilcoxon test. Categorical data were expressed as the relative distribution frequency and percentage using the chi-square test or fisher’s exact test. A *p* value less than 0.05 was regarded as statistical significance. Variables with statistical significance in univariate analysis were fed into multivariable logistic regression analysis with implementing forward stepwise selection method. Kaplan–Meier analysis and the log-rank test were executed to plot the survival curves and to analyze the differences between the curves.

## 3. Results

### 3.1. Clinical Data

**Figure 3:**
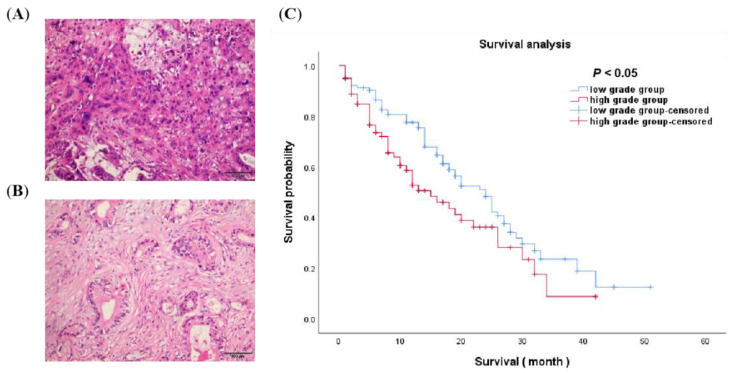
A comparison between patients within the low-grade and the high-grade group. (**A**) High-grade group pathological expression (×20). (**B**) Low-grade group pathological expression (×20). (**C**) The Kaplan–Meier curves of the two groups were significantly different (*p* < 0.05). Patients in the low-grade group had significantly longer survival than those in the high-grade group by log-rank test.

A total of 198 patients were enrolled as the internal training group, including 138 men (age: 60.96 ± 9.85 years; range: 38–85 years) and 60 women (age: 62.17 ± 8.52 years; range: 41–78 years). Moreover, 30 patients were enrolled as the external validation group, including 16 men and 14 women. Detailed baseline characteristics are showed in Table 1. In the internal training group, there are 117 patients in the low-grade cohort and 81 patients in the high-grade cohort; in the external validation group, there are 17 patients in the low-grade group and 13 patients in the high-grade group. Univariate and Multivariable logistic regression analysis showed that gender (statistic= 4.238, *p* = 0.040), T stage (statistic = 9.347, *p* = 0.025), lymph node metastasis (statistics = 10.482, *p* = 0.005) and CA-199 level (statistics = 5.446, *p* = 0.020) were independent predictors of histological grade. The results indicated that the high-grade group were more likely to have male, more higher T stage, lymph node metastasis and abnormal CA-199 level. The Kaplan–Meier curves showed significant difference between low-grade and high-grade PDAC with *p <* 0.05 (Figure 3). An evidently longer survival time was revealed in the low-grade group (mean: 24.19 months, 95% CI: 20.78–27.59) than the high-grade group (median: 17.91 months, 95% CI: 14.37–21.45).

### 3.2. Feature Selection

**Figure 4:**
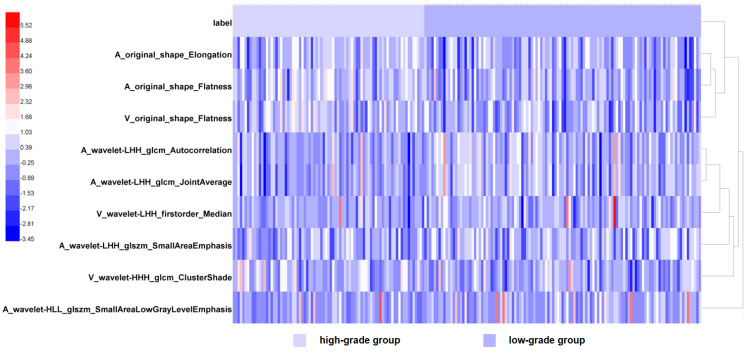
Heatmap of nine radiomics features including. The heatmap is grouped according to the low-grade group and the high-grade group. Each row corresponds to one radiomics feature, and each column corresponds to one patient. The rightmost lines represent hierarchical clustering of radiomics features, shown as a dendrogram.

A total of 960 radiomics features were extracted in every scanning phase; thus, a total of 1920 radiomic features were extracted from every patient. After GBDT algorithm implementation, nine most valuable and important features were selected (Table 2), in which six features are derived from arterial phase and three features are derived from venous phase. GLCM and shape features take up the majority in nine features. Taking the feature types as standard, six features are from wavelet transformation and the remained three are from original images. The features’ heatmap displayed the difference in different groups visually by finding out which two features are most similar and merge them into a cluster, then, repeating this process until all the features are assembled together. Hierarchical clustering is accompanied by the generation of a dendrogram, which shows the feature similarity and order of clustering in this task (Figure 4).

### 3.3. Radiomics Models Evaluation

**Figure 5:**
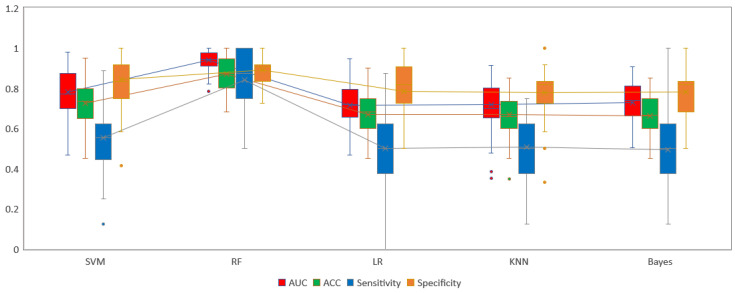
Comparison of five radiomics models based on ten times tenfold cross-validation in internal training group. Box-and-whisker plots of the differences in SVM, RF, LR, KNN and Bayes model among AUC, ACC, Sensitivity and Specificity. RF achieved the best performance in every statistical indicators than other models. The box indicates the 25- and 75- quartile; the horizontal line indicates the median and the cross indicates the mean. Whiskers show the 5- and 95- percentiles; outliers are indicated by circles.

**Figure 6:**
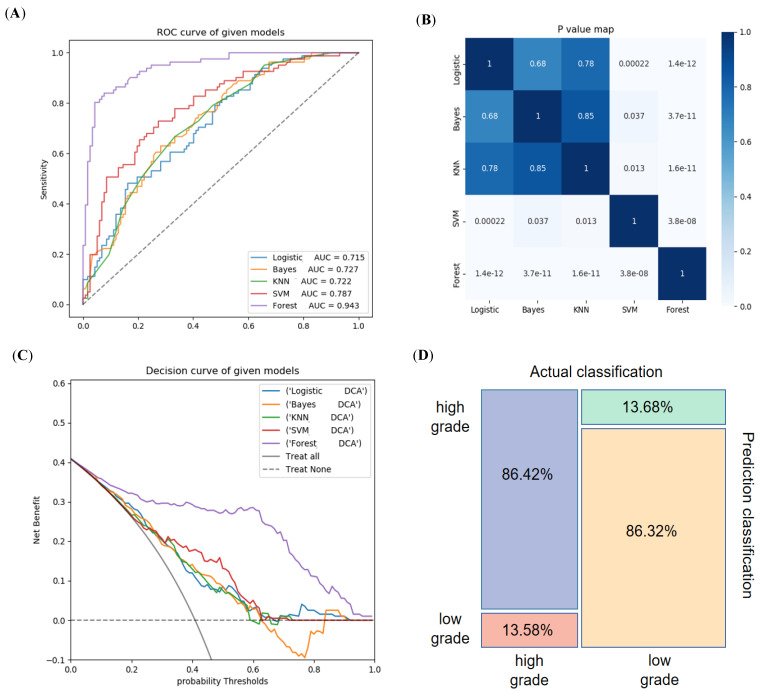
The performance evaluation of the five radiomics models in internal training group. (**A**) The RF model had the maximum AUC, followed by SVM, Bayes, KNN and LR. (**B**) DeLong test showed the AUC of RF model was significantly different than that of other models, followed by SVM model. (**C**) The decision curves show that the RF model had greatest net benefit with a threshold probability > 0.06. (**D**) Mosaic map showed the RF model achieved 86.42% and 86.32% predictive accuracy in the high-grade and the low-grade group, respectively.

The five different machine learning-based models were built including the LR, KNN, Bayes, SVM and RF algorithms via ten times tenfold cross-validation. In internal training group, Figure 5 showed RF achieved the best ability in every statistical indicator. The RF radiomics model reached the optimal performance with an AUC of 0.943 (95% CI, 0.915–0.967) and an accuracy of 0.864, and SVM model achieved the secondary diagnostic ability with an AUC of 0.787 (95% CI, 0.734–0.839) and accuracy of 0.732, followed by the Bayes model, yielding an AUC of 0.727 (95% CI, 0.667–0.784) and accuracy of 0.677. The worst outcome was derived from LR and KNN models between five radiomics model (Figure 6 and Table 3). The delong test demonstrated the AUC of RF model was significantly statistically significant than that of other models (all *p* < 0.001), and the SVM model was also statistically different compared with other four models (all *p* < 0.05). The remaining three models (Bayes, KNN, LR) were not significantly different to each other (*p* > 0.05) (Figure 6B). Figure 6C showed the RF model had the greatest net benefit with a threshold probability > 0.06. One mosaic map using the RF classifier indicated that 70 patients were accurately predicted among 81 patients (86.42%, 70/81) in the high-grade group, and 101 patients (86.32%, 101/117) were accurately predicted among 117 patients in the low-grade group (Figure 6D).

**Figure 7:**
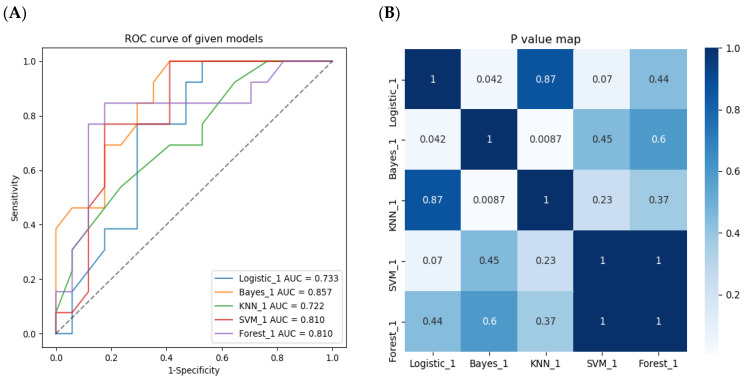
The performance evaluation of the five radiomics models in the external validation group. (**A**)The Bayes model had the maxium AUC, followed by RF model and SVM model. (**B**) Delong test showed there were no significant difference between RF, SVM and Bayes with AUCs each other.

In the external validation group, the result (Table 4 and Figure 7) showed the Bayes achieved the best diagnostic ability with an AUC of 0.857 (95% CI: 0.548–0.912), followed by the RF model with an AUC of 0.810 (95% CI: 0.636–0.984) and SVM model with an AUC of 0.810 (95% CI: 0.648–0.972). There were no significant differences with AUCs between the RF, SVM and Bayes models. The top three models with excellent performance in the external validation group was consistent with that in the internal training group (Bayes, RF, SVM), verifying that our models have stable diagnostic ability and generalization ability. Considering that the RF model have reached outstanding performance both in the internal training group and the external validation group, and the RF model is especially powerful in large data set, RF model is ultimately selected as the best radiomics model to further construct the combined model.

### 3.4. Performance Evaluation of the Clinical and Combined Models

**Figure 8:**
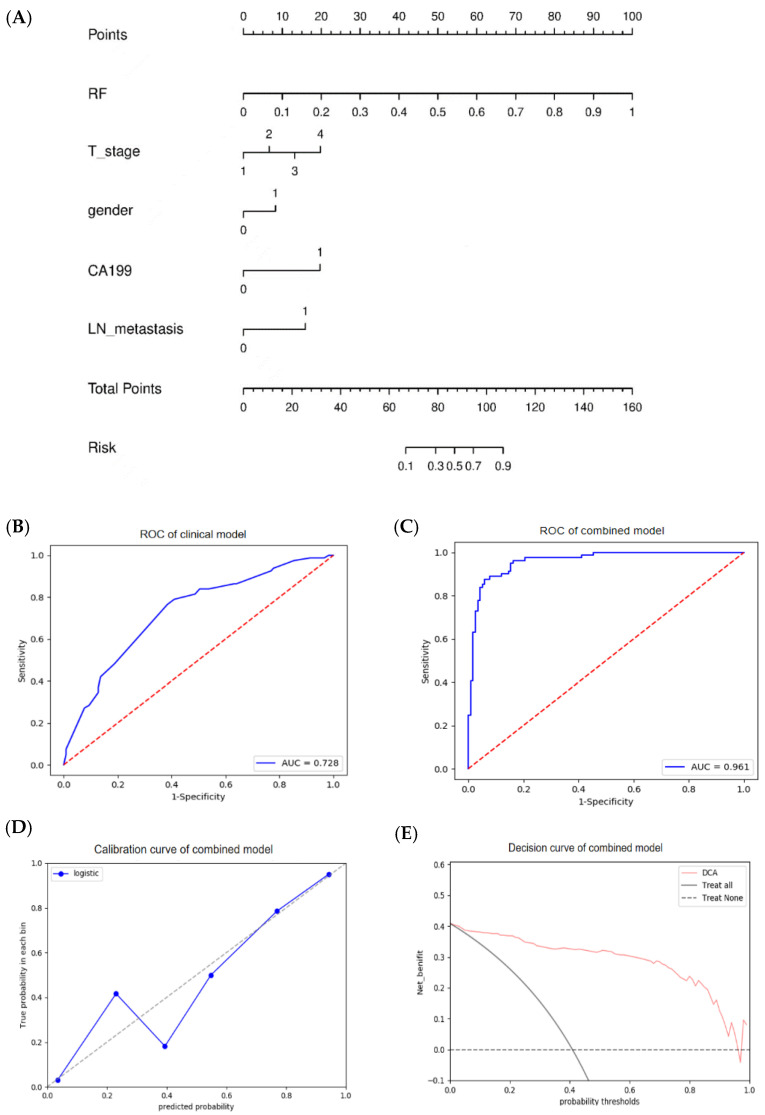
Construction of clinical and combined model in the internal training group. (**A**) A nomogram was visualized by integrating the best radiomics model-RF and screened clinical features including T stage, gender, CA199, lymph node metastasis. The clinical model (**B**) and combined model (**C**) achieved the modest and the best diagnostic performance, respectively. (**D**) The calibration curve of the nomogram presented a good consistency between predicted and observed histological grade. (**E**) The decision curve of the nomogram provided the best diagnostic efficacy over and above the “treat all” or “treat none” scheme.

Due to the fact that the collected samples were relatively small in external validation group, we determined to build clinical and combined model only for internal training group. Meanwhile, we executed multicollinearity check with satisfactory result (Supplementary Table S1). Then, the screened features (gender, T stage, lymph node metastasis, CA-199 level) were built as a clinical model using multivariate logistic regression analysis. The combined model was constructed using the clinical model and RF model, with the highest AUC of 0.961 and an accuracy of 0.909 (Figure 8 and Table 5). The clinical model performed worst with an AUC of 0.728 and an accuracy of 0.677 between RF, clinical and combined model. A nomogram was established as a quantitative diagram for noninvasive LNM prediction (Figure 8A). The calibration curve of the combined model displayed good consistency between the predicted and actual histological grade (Figure 8D). The decision curve demonstrated that the combined model provides the best diagnostic efficacy over and above the “treat all” or “treat none” scheme with a threshold probability between 0.04 and 0.96 (Figure 8E).

## 4. Discussion

In our study, we designed and verified multiple CECT-based radiomics models for differentiating histological grade in PDAC. No matter whether it was the internal training group or the external validation group, the top three (RF, SVM and Bayes) radiomics model achieved satisfactory outcome. Due to the relatively small dataset, according to the TRIPOD criterion, random grouping is not more reliable than the results of total data modeling. In addition, we have performed ten times ten-fold cross-validation to minimize the over-fitting of the model. Thus, we have confidence that our result is reliable and meaningful, and this result could offer an accurate and noninvasive approach to further investigate application value in PDAC evaluation after feasibility of clinical translation. To our knowledge, this is also the first study comparing values of multiple radiomics models for the purpose of preoperatively identifying histological grade in PDAC.

Radiomics, as a noninvasive technology, could investigate and analyze the potential phenotypic message hidden in medical images which is invisible by naked eyes, thereby improving diagnosis and assisting individualized treatment strategy. The radiomics features extracted in our study, mainly included shape features and GLCM features, indicating that high-grade mass grow faster and bigger, and heterogeneity of tumor entity varied more complicatedly and asymmetrical, thus, generating a relationship between a local or overall area of an image and adjacent pixels or pixels within a certain distance which possessed evident difference and variation in the spatial distribution of voxel gray-scale level. Past study reported that CT-based radiomics could discriminate the heterogeneous distribution of neoplastic cells. Less tumor heterogeneity is correlated to lower histological grade [29], which is consistent with our finding. With observing medical images, high-grade PDAC could demonstrate more uneven CT value and easily generate necrosis and microhemorrhage, similarly to Cassinotto [30] who also denoted that poorly differentiated histological grade was relevant to lower central CT value.

Our study demonstrated that the RF, SVM and Bayes models performed better than the LR and KNN models in both internal training group and external validation group. The reasons were speculated that RF as an ensemble learning algorithm is outstanding among current machine learning approaches by integrating multiple decision trees branch node to train the data set based on multiple base classifiers, and the final classification result was obtained by the vote of all base classifiers; Moreover, RF specialize in predicting classification result especially in larger data sets, thus, RF performed the best in internal training group, followed by the SVM model and the Bayes model; and RF performed second in the external validation group. Bayes summed each feature’s predictive probability up which is assumed that features are independent of each other. Bayes behaved particularly well in small data sets. Thus, the classification effect of Bayes is the highest in the external validation group and is modest in the internal training group. This result indicated performance of model depend on algorithm’s characteristics and matching with research objectives. Every experiment should attempt as much as possible models for selecting the best model aiming at different clinical problem. The performance of KNN and LR remained weak in internal training groups and external validation groups. We guessed that this was due to variability and instability of parameters and unfitted with nonlinear or linearly inseparable data.

In this study, integrating the AP and VP features showed the better predictive ability than sole AP or sole VP features in our experiment. Ultimately, the nine most important features were selected including six AP features and three VP features. Some studies [25,31,32] only used one venous phase for the purpose of convenience and regarded VP as a clearer lesion boundary. Our finding implied that AP is significant as well. Thus, enrolling more CT scanning phases might have the opportunity to exploit more complete and abundant inherent characteristics. Chang [25] also used radiomics method to identify histological grade in PDAC, however, it only used one scanning phase (pancreatic parenchymal phase) and one machine learning approach (SVM) and achieving an AUC of 0.910 in the test group and 0.770 in the external validation group. Our research emphasis is quite different from Chang’s. We selected arterial and venous phase, rather than one scanning phase. We compared and investigated multiple radiomics models, rather than only SVM model. We used one novel feature selection method, namely GBDT model, rather than one traditional and outdated LASSO regression model. In addition, the predictive performance of Chang’s study is similar with that of ours in the internal training group, whereas is worse than ours in the external validation group. Thus, we have confidence that our research is valuable and effective and could improve model performance and generalization ability in multicenter study.

Among the clinical risk factors, our study found that the high-grade group were more easily observed males than the low-grade group, which is not consistent with the result of Dunet [26]; we speculated this was due to eating habits of local citizen and races distribution of southeast China. Consistent with our results, Wasif [33] denoted that high-grade group had a significantly higher ratio of clinical T stage and lymph node metastasis, indicating the two features is valuable in predicting worse differentiated degree. Our result indicated that CA19-9 had significant differences for identifying histological grade of PDAC, we guessed this was because CA19-9 is a specific tumor marker which could reflect biological behavior, and the higher CA19-9 is, the poor prognosis patient have. With the consideration of that lymph node metastasis is acquired from postoperative specimens, its diagnostic ability will be further decreased after removing lymph node metastasis. Our combined model integrating aforementioned clinical variables and RF model demonstrated the outstanding diagnostic ability with an AUC of 0.961, which is higher than sole RF model or sole clinical model. It is recommended that despite limitation existed in visual image evaluation, but through fusing multiple clinical information, fusioned models could assess tumor more comprehensively and accurately. Previous studies implied that by adding clinical information, the combined model denoted an evident progress than the radiomics model individually [34]. Thus, clinical information is meaningful and deserving attention in radiomics analysis.

Some limitations exist in our study. First, manual segmentation was subjective. It is essential to realize complete auto segmentation for consistency in the future. Second, this study was based on traditional machine learning methods due to it possess more interpretability and generalization ability without black box theory in deep learning, but deep learning will be attempted in the future. Third, we only focused on arterial and venous phases, and future studies need to integrate more scanning technology, functional or perfusion parameters.

## 5. Conclusions

In summary, among five radiomics models, the preferable performance was achieved by RF, SVM, Bayes in internal and external data groups. Our two-center study demonstrated the combined model integrating with the RF model and clinical features performed greatest, which could represent a precise and noninvasive tool for predicting histological grade of PDAC, thus assisting in clinical decision-making and therapeutic strategies. Acceptable individuals could benefit from early personalized surgical plans.

## Figures and Tables

**Table 1:** Baseline characteristics of patients with PDAC.

Characteristics	Internal Training Group				External Validation Group			
	Low-Grade Group	High-Grade Group	Statistics	*p* Value	Low-Grade Group	High-Grade Group	Statistics	*p* Value
Clinical characteristics								
Age(y), mean ± SD	61.32 ± 9.72	61.33 ± 9.13	−0.010	0.990	60.94 ± 11.48	60.46 ± 8.67	0.126	0.901
Gender			4.238	0.040 *			0.002	0.961
Female	42(35.9)	18(22.2)			8(47.1)	6(46.2)		
Male	75(64.1)	63(77.8)			9(52.9)	7(53.8)		
Abdominal pain			0.386	0.534			0.475	0.491
Yes	70(59.8)	52(64.2)			7(41.2)	7(53.8)		
No	47(40.2)	29(35.8)			10(58.8)	6(46.2)		
Backache			1.186	0.276			1.186	0.276
Yes	24(20.5)	22(27.2)			3(17.6)	2(15.4)		
No	93(79.5)	59(72.8)			14(82.4)	11(84.6)		
Pancreatitis			0.338	0.561			0.136	0.713
Yes	18(15.4)	15(18.5)			2(11.8)	1(7.7)		
No	99(84.6)	66(81.50			15(88.2)	12(92.3)		
Jaundice			0.406	0.524			0.475	0.491
Yes	19(16.2)	16(19.8)			10(58.8)	6(46.2)		
No	98(83.8)	65(80.2)			7(41.2)	7(53.8)		
Operation			1.202	0.273			0.305	0.580
Pancreaticoduodenectomy	86(73.5)	65(80.2)			13(76.5)	11(84.6)		
Distal pancreatectomy	31(26.5)	16(19.8)			4(23.5)	2(15.4)		
Pathological characteristics								
Lymph node metastasis			10.482	0.005 *			4.344	0.037 *
Negative	84(71.8)	41(50.6)			14(82.4)	6(17.6)		
Positive	32(27.4)	40(49.4)			3(17.6)	7(53.8)		
Duodenum Invasion			0.748	0.387			2.330	0.127
Negative	75(64.1)	47(58.0)			7(41.2)	9(69.2)		
Positive	42(35.9)	34(42.0)			10(58.8)	4(30.8)		
Surgical margin status			2.783	0.095			0.305	0.580
Negative	110(94.0)	80(98.8)			13(76.5)	11(84.6)		
Positive	7(6.0)	1(1.2)			4(23.5)	2(15.4)		
Perineural invasion			2.054	0.152			1.639	0.201
Negative	20(17.1)	8(9.9)			2(11.8)	0(0.0)		
Positive	97(82.9)	73(90.1)			15(88.2)	13(100)		
Imaging characteristics								
CT-reported tumor size(mm)	28.03 ± 11.02	29.11 ± 11.30	−0.669	0.504	28.65 ± 9.27	31.31 ± 9.93	−0.755	0.456
Location			1.726	0.422			0.679	0.410
Head and neck	88(75.2)	66(81.5)			15(88.2)	10(76.9)		
Body and tail	29(24.8)	15(18.5)			2(11.8)	3(23.1)		
Tumor density			0.772	0.680			-	-
Hypodensity	114(97.4)	80(98.8)			17(100)	13(100)		
Isodensity	2(1.7)	1(1.2)			0(0.0)	0(0.0)		
Hyperdensity	1(0.9)	0(0.0)			0(0.0)	0(0.0)		
T stage			9.347	0.025 *			2.90	0.235
cT1	30(25.6)	9(11.1)			3(17.6)	0(0.0)		
cT2	72(61.5)	55(67.9)			12(70.6)	10(76.9)		
cT3-4	15(12.8)	17(21.0)			2(11.8)	3(23.1)		
Metastasis			0.140	0.709			0.084	0.773
cM0	115(98.3)	79(97.5)			15(88.2)	11(84.6)		
cM1	2(1.7)	2(2.5)			2(11.8)	2(15.4)		
Parenchymal atrophy			0.003	0.958			0.136	0.713
Yes	64(55.2)	45(55.6)			3(17.6)	3(23.1)		
No	52(44.8)	36(44.4)			14(82.4)	10(76.9)		
PD dilatation			0.121	0.728			0.679	0.410
Yes	90(76.9)	64(79.0)			15(88.2)	10(76.9)		
No	27(23.1)	17(21.0)			2(11.8)	3(23.1)		
CBD dilatation			0.062	0.803			0.197	0.657
Yes	76(65.0)	54(66.7)			4(23.5)	4(30.8)		
No	41(35.0)	27(33.3)			13(76.5)	9(69.2)		
Laboratory characteristics								
CA-199 level			5.446	0.020 *			0.151	0.697
Normal	34(29.1)	12(14.9)			5(29.4)	3(23.1)		
Abnormal	83(70.9)	69(85.2)			12(70.6)	10(76.9)		
CEA level			0.001	0.972			0.197	0.657
Normal	98(83.8)	68(84.0)			13(76.5)	9(69.2)		
Abnormal	19(16.2)	13(16.0)			4(23.5)	4(30.8)		
TBIL level			0.338	0.561			0.362	0.547
Normal	54(46.2)	34(42.0)			6(35.3)	6(46.2)		
Abnormal	63(53.8)	47(58.0)			11(46.2)	7(53.8)		

Note: * highlights the *p* values that are smaller than 0.05. Categorical data are number of patients; data in parentheses are percentage. Abbreviation:PD, pancreatic duct; CBD, common bile duct; CA 19–9, carbohydrate antigen 19–9; CEA, carcino-embryonic antigen; TBIL, total bilirubin.

**Table 2:** Radiomics features’ selection results.

CT Scanning Phase	ID	Radiomics Features’ Name
Arterial phase	1	original_shape_Elongation
	2	original_shape_Flatness
	3	wavelet-LHH_glcm_Autocorrelation
	4	wavelet-LHH_glcm_JointAverage
	5	wavelet-LHH_glszm_SmallAreaEmphasis
	6	wavelet-HLL_glszm_SmallAreaLowGrayLevelEmphasis
Venous phase	1	original_shape_Flatness
	2	wavelet-LHH_firstorder_Median
	3	wavelet-HHH_glcm_ClusterShade

**Table 3:** Model performance of five radiomics models in internal training group.

Model	AUC	Sensitivity	Specificity	Accuracy	f1_Score	Recall
LR	0.715 (0.658–0.772)	0.469	0.838	0.687	0.551	0.469
KNN	0.722 (0.664–0.779)	0.667	0.667	0.667	0.621	0.667
Bayes	0.727 (0.667–0.784)	0.58	0.744	0.677	0.595	0.58
SVM	0.787 (0.734–0.839)	0.556	0.855	0.732	0.629	0.556
RF	0.943 (0.915–0.967)	0.864	0.863	0.864	0.838	0.864

Note. Numbers in parentheses are the 95% confidence interval. Abbreviation: logistic regression (LR), k-nearest neighbor (KNN), naive bayes model (Bayes), support vector machine (SVM), random forest (RF), areas under receiver operating characteristic curve (AUC).

**Table 4:** Comparison of radiomics model performance in the external validation group.

Models	AUC	95%CI
LR	0.733	0.548–0.912
Bayes	0.857	0.727–0.988
KNN	0.722	0.538–0.905
SVM	0.810	0.648–0.972
RF	0.810	0.636–0.984

Abbreviation: logistic regression (LR), k-nearest neighbor (KNN), naïve bayes model (Bayes), support vector machine (SVM), random forest (RF), areas under receiver operating characteristic curve(AUC).

**Table 5:** Model performance of the best radiomics model, clinical model and combined model in internal training group.

Model	AUC	Sensitivity	Specificity	Accuracy	f1_Score	Recall
RF	0.943 (0.915–0.967)	0.864	0.863	0.864	0.838	0.864
Clinical	0.728 (0.667–0.790)	0.481	0.812	0.677	0.549	0.481
Combine	0.961 (0.938–0.980)	0.864	0.940	0.909	0.886	0.864

Abbreviation: random forest (RF).

## Data Availability

The datasets used and/or analyzed during the current study are available from the corresponding author upon reasonable request.

## Supplementary Materials

The following supporting information can be downloaded at: https://www.mdpi.com/article/10.3390/diagnostics12081915/s1, Table S1: Multicollinearity check. Relevant references are [24,35,36].
