# Supplementary material for: Comparison of Multiple Radiomics Models for Identifying Histological Grade of Pancreatic Ductal Adenocarcinoma Preoperatively Based on Multiphasic Contrast-Enhanced Computed Tomography: A Two-Center Study in Southwest China
Source: Diagnostics (Basel). 2022 Aug 8;12(8):1915. doi: 10.3390/diagnostics12081915 (PMC9406915; doi:10.3390/diagnostics12081915)
Supplement: Supplementary file 1 [file diagnostics-12-01915-s001.zip › diagnostics-1838922-supplementary.pdf]

Supplementary Materials

Comparison of Multiple Radiomics Models for Identifying Histological Grade of Pancreatic Ductal Adenocarcinoma Preoperatively Based On Multiphasic Contrast-Enhanced Computed Tomography: A Two-Center Study in Southeast China

The definitions of the clinical features are listed as follows:

(1) clinical characteristics were recorded from electronic medical record

gender, age, abdominal pain, backache, history of pancreatitis, jaundice, operation method

(2) pathological characteristics were recorded from pathological report

lymph node metastasis, duodenal invasion, surgical margin status, perineural invasion

(3) imaging characteristics

1.CT-reported tumour size(mm): the maximum tumour diameter in cross section

2.tumour location: head and neck, body and tail

3.tumour density (hypodensity, isodensity, hyperdensity): Referring relevant literature [1], we defined tumor density as the difference between the attenuation of tumor and normal pancreatic phase in arterial and venous phase. If normal pancreatic phase density minus tumor density, delta > 20, it represent hypodensity; if 0 < delta < 20 , it represent isodensity; if delta < 0, it represent hyperdensity. Tumor-pancreas contrast was calculated for each patient by drawing ROIs for both tumor and normal pancreas. Care was taken to exclude macroscopic enhanced blood vessels and pancreatic duct from the ROI.

4.clinical T stage was assessed using CT-reported tumour size according to the AJCC TNM Staging System Manual, 8th Edition [3].

5.distant metastasis: there were visible organ or tissue metastasis

6.focal parenchymal atrophy: FPA is defined as narrowing of the focal parenchyma in comparison to both the head- and tail-side parenchyma, showing a cave-in, slim, or slit-like appearance[2].

7.pancreatic duct dilatation: we define pancreatic duct dilatation as >3 mm

8.common bile duct dilatation: we define common bile duct dilatation as >10 mm

(4) laboratory characteristics:

1.carcino-embryonic antigen (CEA) level: abnormal CEA as > 5 ng/ml

2.carbohydrate antigen 19-9 (CA19-9) level : abnormal CA199 > 37 U/ml

3.total bilirubin (TBIL) level: abnormal TBIL level as > 20.5μmol/L

Reference:

[1] Prokesch, R. W., Chow, L. C., Beaulieu, C. F., Bammer, R., & Jeffrey, R. B., Jr (2002). Isoattenuating pancreatic adenocarcinoma at multi-detector row CT: secondary signs. Radiology, 224(3), 764–768. https://doi.org/10.1148/radiol.2243011284

[2] Nakahodo J., Kikuyama M., Nojiri S., Chiba K., Yoshimoto K., Kamisawa T., Horiguchi S.I., Honda G. Focal Parenchymal Atrophy of Pancreas: An Important Sign of Underlying

High-Grade Pancreatic Intraepithelial Neoplasia Without Invasive Carcinoma, i.e., Carcinoma in Situ. Pancreatology. 2020;20:1689–1697. doi: 10.1016/j.pan.2020.09.020.
[3] Chun Y, Pawlik T,Vauthey J. (2018) 8th Edition of the AJCC Cancer Staging Manual: Pancreas and Hepatobiliary Cancers. Annals of surgical oncology, 25(4),845–847. doi:10.1245/s10434-017-6025-x

Table S1 Multicollinearity check

Coefficients[a]

Model		Collinearity Statistics	
		Tolerance	VIF
1	(Constant)		
	CT-reported tumor size	0.215	4.655
	CT-reported T stage	0.229	4.372
	age	0.879	1.138
	gender	0.872	1.147
	abdominal pain	0.789	1.267
	backache	0.847	1.180
	history of pancreatitis	0.906	1.104
	jaundice	0.880	1.136
	CEA	0.859	1.164
	CA199	0.860	1.162
	TBIL	0.559	1.788
	tumor density	0.937	1.068
	distant metastasis	0.826	1.211
	parenchymal atrophy	0.798	1.253
	pancreatic duct dilatation	0.671	1.489
	CBD dilation	0.424	2.358
	duodenal invasion	0.719	1.391
	lymph node metastasis	0.840	1.191
	surgical margin status	0.880	1.136
	perineural invasion	0.745	1.342
	tumor location	0.445	2.246
	operation	0.293	3.407

a. Dependent Variable: histological grade

Note:A total of 22 features achieved no multicollinearity with tolerance > 0.1 and VIF < 5.
